# Antivirals and vaccines for Enterovirus A71

**DOI:** 10.1186/s12929-019-0560-7

**Published:** 2019-09-03

**Authors:** Jing-Yi Lin, Yu-An Kung, Shin-Ru Shih

**Affiliations:** 1 Department of Clinical Laboratory Sciences and Medical Biotechnology, College of Medicine, National Taiwan University, Taipei City, Taiwan; 2 Research Center for Emerging Viral Infections, College of Medicine, Chang Gung University, Taoyuan, Taiwan; 3 Department of Medical Biotechnology and Laboratory Science, College of Medicine, Chang Gung University, Taoyuan, Taiwan; 4 Department of Laboratory Medicine, Linkou Chang Gung Memorial Hospital, Taoyuan, Taiwan; 5 Research Center for Chinese Herbal Medicine, Research Center for Food and Cosmetic Safety, and Graduate Institute of Health Industry Technology, College of Human Ecology, Chang Gung University of Science and Technology, Taoyuan, Taiwan

**Keywords:** Enterovirus A71, Antivirals, Vaccines

## Abstract

Enterovirus A71 (EV-A71) is an important emerging virus posing a threat to children under five years old. EV-A71 infection in infants or young children can cause hand-foot-and-mouth disease, herpangina, or severe neurological complications. However, there are still no effective antivirals for treatment of these infections. In this review, we summarize the antiviral compounds developed to date based on various targets of the EV-A71 life cycle. Moreover, development of a vaccine would be the most effective approach to prevent EV-A71 infection. Therefore, we also summarize the development and clinical progress of various candidate EV-A71 vaccines, including inactivated whole virus, recombinant VP1 protein, synthetic peptides, viral-like particles, and live attenuated vaccines.

## Background

Enterovirus A71 (EV-A71), a positive-strand RNA virus of the family *Picornaviridae*, represents a persistent global health problem and has caused large outbreaks in the Asia-Pacific region in recent years [1]. Infection by EV-A71 can result in hand-foot-and-mouth disease (HFMD) and herpangina. Children under five years old are particularly susceptible to the most severe forms of EV-A71-associated neurological complications, including aseptic meningitis, brainstem and/or cerebellar encephalitis, myocarditis, acute flaccid paralysis, and rapid fatal pulmonary edema and hemorrhage [2]. Owing to the lack of effective drugs for inhibiting EV-A71 infection, supportive therapy remains the primary means of managing severe cases. Nevertheless, there has been substantial progress in identifying candidate targets for anti-viral drugs and vaccines.

The enterovirus genome encodes four structural capsid proteins (VP1, VP2, VP3, and VP4) that facilitate the cellular entry and delivery of the viral genome into the cytosol of the host cell, and seven non-structural proteins (2A^pro^, 2B, 2C, 3A, 3B, 3C^pro^, and 3D^pol^) that mediate viral RNA replication [3]. Antiviral therapy and vaccines can have a variety of mechanisms of action and each step of the virus replication cycle can be targeted. Here, we summarize these recent advances and some of the key compounds showing potential for new therapeutic strategies in the development of vaccine and antiviral compounds that target the life cycle of EV-A71, and discuss the prospects and challenges in this field.

## Inhibitors of the EV-A71 life cycle

### Capsid inhibitors

The first step in successful viral infection is receptor binding, and the capsid protein VP1 is involved in the recognition of EV-A71 receptors on the surface of host cells. Numerous viral receptors that are responsible for the entry of EV-A71 into host cells have been characterized, including human scavenger receptor class B member 2 (hSCARB2) [4], human P-selectin glycoprotein ligand 1 (PSGL-1) [5], annexin A2 (Anx2) [6], heparan sulfate [7], sialylated glycan [8], and dendritic cell-specific intercellular adhesion molecule-3 grabbing nonintegrin (DC-SIGN) [9]. Various approaches have been proposed for the discovery of antivirals targeting EV-A71 host receptor binding.

Antibodies against SCARB2, PSGL-1, and DC-SIGN effectively inhibited EV-A71 infection in a dose-dependent manner [4, 5, 10, 11]. EV-A71 VP1 inhibitors were the first candidates proposed for developing antivirals against viral infection. To date, pleconaril and vapendavir have been identified to bind to the viral capsid and thus inhibit EV-A71 infection [12]. Moreover, the capsid binder pyridyl imidazolidinone showed notable potency against EV-A71 infection in several consecutive studies [13–15]. Pyridyl imidazolidinone fits into the viral hydrophobic pocket of VP1 to inhibit viral infection [16, 17]. In addition, an imidazolidinone derivative (PR66) was found to inhibit EV-A71 infection by impeding the uncoating process via its interaction with the capsid protein VP1. PR66 could also protect against EV-A71-induced neurological symptoms in vivo by suppressing EV-A71 replication [18].

One compound (14) of aminopyridyl 1,2,5-thiadiazolidine 1,1-dioxides, which was capsid inhibitor, showed anti-EV-A71 infection effects (EC_50_ = 4 nM) and exhibited good in vivo efficiency in an EV-A71-infected mouse model [19]. A sulfonated food azo dye, Brilliant Black BN (E151), was reported to inhibit EV-A71 infection by competing with EV71 attachment factors for viral binding, thereby blocking viral attachment/entry to host cells in vitro; moreover, in vivo studies demonstrated that daily administration of E151 at 200 mg/kg given in the initial four days of challenge protected AG129 mice challenged with a 10 of 50% lethal dose (LD_50_) of EV-A71 [20].

### 2A^pro^ inhibitors

2A^pro^ is enterovirus-encoded protease ad is important for viral polyprotein processing [21, 22]. 2A^pro^ could also cleave the host factor eIF4GI to inhibit the cap-dependent translation of cellular mRNA. Falah and coworkers showed that a six-amino acid peptide (LVLQTM) exhibited antiviral potencies against EV-A71 in HeLa cells. This peptide bound to the 2A^pro^ active site and inhibited eIF4GI cleavage by 2Apro [23].

### 2B inhibitor

Viral protein 2B and its precursor 2 BC have been suggested to be responsible for membranous alteration and inhibition of cellular protein secretion in infected cells [24, 25]. EV-A71 2B protein also induced cell apoptosis by modulating Bax protein activation [26]. Xie and colleagues reported that 4,4′-diisothiocyano-2,2′-stilbenedisulfonic acid (DIDS), which is a chloride-dependent current inhibitor, could prevent EV-A71 2B activity and lead to the inhibition of virus production in RD cells [27].

### 2C inhibitor

The 2C protein plays a role in viral replication complex formation and is involved in processing nucleoside triphosphatase activity and in the synthesis of RNA-negative strands [28, 29]. Two adenosine analogs, metrifudil and N6-benzyladenosine, have been demonstrated to interact with 2C protein to inhibit EV-A71 infection [30].

### 3A inhibitor

The 3A protein also plays a role in viral replication complex formation and inhibits cellular protein secretion. An enviroxime mimetic compound, AN-12-H5, was found to inhibit EV-A71 infection. Assays with resistant mutants have suggested that AN-12-H5 blocked replication by targeting 3A and also inhibited an early stage of infection by targeting VP1 and VP3 [31]. Another compound, GW5074, 3-(3,5-dibromo-4-hydroxybenzylidine-5-iodo-1,3-dihydro-indol-2-one), which is a Raf-1 inhibitor, has been demonstrated to target 3A to inhibit EV-A71 infection [30, 32].

### 3C^pro^ inhibitor

The 3C protein acts as a protease to cleave viral polypeptides toward their conversion to mature viral proteins during viral infection, and is thus another useful target for antiviral therapy. The compound rupintrivir (also known as AG7088) inhibited EV-A71 3C protein by mimicking the substrate of the 3C protein in vitro and protected suckling mice from EV-A71-caused limb paralysis in vivo [33, 34]. On the other hand, a series of rupintrivir analogues have also been synthesized and more inhibited EV-A71 3C protease activity and viral growth than rupintrivir [35]. Another 3C protease inhibitor (SG85) also inhibited the replication of 21 EV-A71 strains [12].

Cyanohyfdrin (R)-1 is another potent inhibitor of EV-A71 3C^pro^ but was unstable and showed potential toxicity. Modifying the labile cyanohydrin moiety led to the discovery of the 4-iminooxazolidin-2-one-based inhibitors 4e and 4 g with potent inhibitory activity and significantly improved stability [36]. One small-molecule inhibitor, DC07090, inhibited EV-A71 replication with an EC_50_ value of 22.09 ± 1.07 μM by targeting 3C protease [37]. Luteoloside is a member of the flavonoids family that exhibits several bioactivities, including anti-microbial and anti-cancer activities, and was also shown to act as a 3C protease inhibitor of EV-A71 in vitro [38].

### 3D^pol^ inhibitor

3D^pol^ of EV-A71 is an RNA-dependent RNA polymerase that plays a role in viral RNA synthesis. DTriP-22 is a non-nucleoside analogue that was shown to inhibit EV-A71 infection by reducing the accumulation of viral RNA [39]. Aurintricarboxylic acid, a compound of a group of polyanionic compounds, could also prevent EV-A71 infection through interference with 3D^pol^ in vitro [40]. As another antiviral strategy, monoclonal antibodies against EV-A71 3D^pol^ were generated to inhibit polymerase activity and viral replication [41].

Apolipoprotein B messenger RNA-editing enzyme catalytic polypeptide-like 3G (APOBEC3G or A3G) can interact with viral 3D^pol^ and viral RNA and can be packaged into progeny virions to reduce the infectivity. APOBEC3G is also a mediator of the antiviral activity of IMB-Z, an *N*-phenylbenzamide derivative [42].

### Viral release inhibitor

Retro-2^cycl^ and Retro-2.1 are inhibitors of several pathogens specifically targeting intracellular vesicle transport, and also participate in EV-A71 life cycle processes, including progeny virus release in vitro. Administration of Retro-2^cycl^ at 10 mg/kg significantly protected 90% of newborn mice from lethal EV-A71 challenge [43].

### Internal ribosome entry site (IRES) inhibitor

The 5′ untranslated region (UTR) of the EV-A71 genome is about 745 nucleotides long and highly structured, containing a cloverleaf-like structure that is critical for viral RNA synthesis and an IRES that is important for viral translation. Idarubicin (IDR) is an anthracycline compound and a USA Food and Drug Administration-approved anticancer drug. IDR inhibits EV-A71 through impaired binding between the EV-A71 IRES RNA and hnRNP A1, a known host IRES *trans*-acting factor [44].

## Other strategies targeting EV-A71

### Ribavirin

Ribavirin is a nucleotide analogue that can serve as a base analogue of either ATP or GTP, and was reported to reduce the EV-A71 titer in vitro. Ribavirin also significantly reduced the mortality, morbidity, and subsequent paralysis sequelae in EV-A71-infected mice [45, 46].

### RNA interference

RNA interference, a native and specific post-transcriptional gene silencing mechanism, has also been exploited as another antiviral tool against EV-A71 infection in vitro and in vivo*.* Short hairpin RNA (shRNA) expression plasmids or small interfering RNAs (siRNAs) that specifically targeted viral genome to inhibit viral protein expression and viral infection [47–52].

### MicroRNA (miRNA)

MiRNAs are approximately 19–24-nucleotide-long non-coding RNAs that post-transcriptionally repress gene expression by targeting mRNAs, and play a pivotal role in the complicated interaction networks between viruses and their hosts. MiRNAs regulate viral replication through multiple mechanisms. For example, miR-9-5p was shown to exert an anti-EV-A71 effect in cells and in a mouse model via mediating the nuclear factor-kappa B (NF-κB) activity of the RIG-I signaling pathway [53]. In addition, miR-2911 inhibited EV-A71 replication via targeting the VP1 gene [54]. MiR-23b could also inhibit EV-A71 replication through downregulation of EV-A71 VPl protein [55]. Overexpression of miR-16-5p enhanced EV-A71-induced apoptosis and inhibited viral replication [56]. MiR-134 inhibited both EV-A71 and poliovirus infection [57], and miR-27a suppressed EV-A71 replication by directly targeting the epidermal growth factor receptor gene [58]. The human miRNA hsa-miR-296-5p suppressed EV-A71 replication by targeting the viral genome located in the regions of nt 2115 to 2135 and nt 2896 to 2920 (strain BrCr) [59]. These studies provide novel mechanisms for the miRNA-mediated regulation of EV-A71 in host cells, suggesting a novel approach in combating infection and in the development of antiviral strategies.

### Heparan sulfate (HS) mimetics

HS is present in the extracellular matrix, on cell surfaces, and in the intracellular granule secretions of all types of animal tissues. HS mimetics are a group of soluble synthetic or semi-synthetic compounds that are structurally related to cellular HS, and can stimulate the functions of cell-surface HS. HS is also a receptor of EV-A71. HS mimetics exhibited anti-EV-A71 activity at less than 250 mg/ml in Vero cells [60].

### Signal pathway targets

GS-9620, a potent and selective agonist of Toll-like receptor 7, could inhibit EV-A71 replication mainly through the NF-κB and PI3K-AKT signaling pathways [61]. Berberine inhibited EV-A71 replication by downregulating autophagy and the MEK/ERK signaling pathway [62]. Isochlorogenic acid C showed antioxidant activity and prevented EV-A71 infection by modulating the redox homeostasis of glutathione [63].

## Development of an EV-A71 vaccine

### Inactivated whole EV-A71 vaccine

Vaccination is considered to be one of the most effective ways to protect against virus infection. Although there are many different approaches available for developing EV-A71 vaccines, including inactivating the whole virus, a live attenuated virus, virus-like particles (VLPs), recombinant subunits, and synthetic peptides, currently, only an inactivated whole virus vaccine for EV-A71 is the only candidate that has proceeded to a completed human clinical trial. To date, inactivated whole EV-A71 vaccines have been established in Taiwan, China, and Singapore. Three vaccine organizations, including Beijing Vigoo Biological Co., Ltd. (Vigoo), Sinovac Biotech Co., Ltd. (Sinovac), and the Chinese Academy of Medical Sciences (CAMS) in China completed EV-A71 vaccine phase III clinical trials in 2013 and received a license for their administration that was approved by China’s Food and Drug Administration in 2015 [64, 65].

These three vaccine organizations in China used different technologies to develop an EV-A71 vaccine. CAMS used KMB-17 human diploid cells as a cell bank that were cultured using a cell factory, whereas Vigoo and Sinovac used Vero cells to amplify EV-A71 with a microcarrier bioreactor and a cell factory, respectively. All organizations selected the EV-A71 C4 subgenotype as a virus seed for vaccine development, which is the most prevalent genotype circulating in China, although they each used a different virus strain: CAMS chose the EV-A71 FY-23 strain, Vigoo chose the FY7VP5 strain, and Sinovac chose the H07 strain. The three organizations began their phase I clinical trials in 2010 to 2011, and completed their phase III clinical trials in 2013. In the Vigoo phase III clinical trial, a total of 10,245 participants aged 6–35 months randomly received a 320 U (EV-A71 antigen unit) alum-adjuvant vaccine (5120 participants) or a placebo control (5125 participants) at days 0 and 28, and were then followed-up for 1 [66] and 2 years [67] (ClinicalTrials.gov, number NCT01508247). The efficacy of the Vigoo EV-A71 vaccine against EV-A71-associated HFMD was 90%, and that against other EV-A71-associated diseases was 80.4% during the 1-year surveillance period. In addition, the vaccine efficacy against EV-A71-associated HFMD was 100% during the second year, and no serious adverse events were reported. Thus, Vigoo claimed that their EV-A71 vaccine is safe and had good efficacy for protecting against EV-A71-associated HFMD in children. Sinovac also conducted a follow-up study for 1 and 2 years [68, 69] in which a total of 10,077 participants aged 6–35 months were assigned to two groups receiving 400 U of the alum-adjuvant Sinovac EV-A71 vaccine or a placebo control at days 0 and 28 (ClinicalTrials.gov, number NCT01507857). During the 1-year surveillance period, the vaccine efficacy was 94.8% against EV-A71-associated HFMD or herpangina, and was 100% against EV-A71-associated HFMD with neurological complications. Given this success, they extended their study to follow-up the vaccine efficacy for another 12 months [69], and reported a vaccine efficacy of 95.1% for the second year; the overall efficacy of the Sinovac EV-A71 vaccine against EV-A71-associated HFMD was 94.7% [68]. Recently, a five-year follow-up study also indicated that the Sinovac EV-A71 vaccine showed long-term immunity persistence [70]. In the phase III clinical trial of the CAMS EV-A71 vaccine, 12,000 children of 6–71 months of age were assigned (at a 1:1 ratio) to receive 100 U of the alum-adjuvant vaccine or placebo control (ClinicalTrials.gov number, NCT01569581). The vaccine efficacy against EV-A71-associated HFMD was 97.4% [71]. However, both the Sinovac and CAMS EV-A71 vaccines showed no efficacy against HFMD caused by coxsackievirus A16 (CV-A16), demonstrating their specificity [68, 71].

In contrast to these three organizations in China, the National Health Research Institutes (NHRI) in Taiwan used the EV-A71 clinical isolate E59 strain (B4 subgenotype) as a virus seed, which was grown in Vero cells cultured with roller-bottle technology. This strain was chosen for producing the EV-A71 inactivated vaccine because of its confirmed genetic stability over several passages and its ability to grow well in Vero cells [72]. The phase I clinical trial of the NHRI was completed in 2012 (ClinicalTrials.gov number, NCT01268787). Sixty heathy adults aged 20–60 years randomly received two intramuscular doses of either 5 μg of EV71 antigen with 150 μg of aluminum adjuvant or 10 μg of EV71 antigen with 300 μg of aluminum adjuvant, 21 days apart. The immunogenicity results indicated that the EV-A71 vaccine produced from the NHRI was safe and immunogenic in healthy adults [73]. Moreover, over 85% of the participants developed a strong cross-neutralizing antibody response against subgenotypes B1, B5, and C4a; however, only 20% of the participants developed a weak cross-neutralizing antibody response against subgenotype C4b and CV-A16 [74]. Two organizations of Taiwan, Enimmune Corp. and Medigen Vaccinology Corp., continue to evaluate the safety and immunogenicity of the E59 strain EV-A71 vaccine in phase II clinical trials (ClinicalTrials.gov number, NCT02777411, NCT03268083 and NCT02200237). In the clinical trial of Medigen Vaccinology Corp., a total of 365 infants or children aged 2 months to 11 years received different doses (low, mid, or high) of alum-adjuvant EV-A71 vaccine or the placebo control in a double-blind and randomized design (ClinicalTrials.gov number, NCT02200237). No vaccine-related serious adverse events were reported in this trial. In addition, the EV-A71 vaccine could elicit an immune response against not only subgenotype B4 but also B5, C4a, C4b, and C5. The EV-A71 vaccine also showed persistence for 2 years [75]. Based on these findings, Medigen Vaccinology Corp. is initiating a phase III clinical trial (ClinicalTrials.gov number, NCT03865238) in 2019, which is expected to be completed in 2022.

In Singapore, Inviragen Inc. (Takeda Pharmaceuticals International, Inc.) completed a phase I clinical trial of an EV-A71 vaccine in April 2012 (ClinicalTrials.gov number, NCT01376479). In contrast to the organizations of China and Taiwan, Inviragen used the B3 subgenotype as the virus seed for EV-A71 vaccine production, which was named INV21. A total of 36 adults aged 21–45 years received two doses (low or high) of INV21 or placebo control 28 days apart. Inviragen claimed that INV21 induced a high immune response against HFMD caused by EV-A71. However, there has been no further clinical trial conducted in Singapore recently.

### Recombinant VP1 vaccine

VP1 is not only a structural protein of EV-A71 but also exhibits strong antigenicity. Accordingly, several research groups have adopted various strategies to express EV-A71 VP1. Wu et al. [76] produced recombinant VP1 proteins of EV-A71 expressed by *Escherichia coli* (*E. coli*). The purified VP1 proteins were then injected into adult female mice through an intraperitoneal route. Although the VP1 subunit vaccine could protect suckling mice against a lower challenge dose of EV-A71 (230 LD_50_ virus/mouse), the inactivated EV-A71 vaccine still elicited a greater immune response than the VP1 subunit vaccine and protected suckling mice against a lethal dose (2300 LD_50_ virus/mouse) of EV-A71. Zhou et al. [77] also expressed recombinant VP1 protein in *E. coli*, and then vaccinated rabbits with the purified VP1 protein or heat-inactivated EV-A71 virus, which elicited comparable humoral and cellular immune responses. Moreover, maternal antibodies protect newborn mice against EV-A71 challenge. EV-A71-specific antibodies of immunized mice were elicited by purified recombinant baculovirus expressing VP1. In addition, the antisera exhibited cross-neutralization activities against different subgenotypes of EV-A71 [78]. Wang et al. [79] generated an HIV-gag-based VLP as a carrier to express EV-A71 VP1 protein, which provided passive protection of newborn mice against EV-A71 infection.

VP1 protein has also been developed as an antigen for oral vaccine development. Adult female BALB/c mice were orally immunized with transgenic tomato fruit expressing VP1 protein [80], attenuated *Salmonella enterica* serovar Typhimurium expressing VP1 [81], VP1-expressing *Bifidobacterium longum* [82], surface-displayed VP1 *Saccharomyces cerevisiae* [83], or recombinant *Lactococcus lactis* expressing secretory VP1 [84]. All of these VP1-expressing vaccines elicited immune responses by oral immunization and could protect newborn mice against EV-A71 infection. Chen et al. [85] generated a transgenic mouse that can express VP1 and secrete into their milk, which could protect suckling mice against EV-A71 challenge. However, the recombinant VP1 proteins generally exhibited lower protective efficacy in mice compared to the inactivated EV-A71 virus.

### Synthetic peptide vaccines

Synthetic peptides have also been tested as an alternative strategy to develop EV-A71 vaccines, which are considered to be safe and efficacious for multivalent vaccines development. The majority of research related to antigen peptides has focused on mapping EV-A71 structural proteins (VP1, VP2, VP3, and VP4). Initially, Foo et al. [86] found that two peptides, SP55 (amino acids 163–177 of VP1) and SP70 (amino acids 208–222 of VP1), could elicit neutralizing antibodies against EV-A71. SP70 elicited a higher titer of neutralizing antibody (1:32) than the neutralizing antibody of SP55 (1:8); however, antisera from heat-inactivated EV-A71-immunized mice elicited the highest neutralization titer of 1:128 [86]. Moreover, Foo et al. [87] found that anti- SP70 antisera passively protected suckling mice against both homologous and heterologous EV-A71 strains. In another strategy, six synthetic peptides (P_70–159_ in VP2, P_140–249_ in VP2, P_324–443_ in VP2, and P_746–876_ in VP1) were combined, which induced the antisera and passively protected newborn mice against EV-A71 infection [88]. The synthetic peptide VP2–28 (amino acids 136–150 of VP2) showed cross-neutralizing activity against EV-A71 and can bind to the anti-EV-A71 monoclonal antibody MAB979 [89]. Xu et al. [90] generated a fusion protein with hepatitis B virus core protein (HBc) and VP2 epitope corresponding to amino acids 141–155 of VP2, named HBc-VP2 (aa141–155), which induced cross-neutralizing EV-A71 antibodies, and the anti-sera from HBc-VP2 (aa141–155) immunized mice protected newborn mice from EV-A71 infection. Huo et al. [91] used the same strategy to display EV-A71 epitopes (SP70, amino acids 208–222 of VP1) and CV-A16 (PEP91, amino acids 271–285 of VP1) using HBc as a carrier protein. The chimeric VLP expressing SP70 and PEP91 epitopes induced an immune response and protected suckling mice against both EV-A71 and CV-A16 infection. Aw-Yong et al. [92] sought to comprehensively map the potential synthetic peptides within the structural and non-structural proteins of EV-A71. A total of 63 synthetic peptides were synthesized and used for characterization of EV-A71 B-cell linear epitopes. Among these, synthetic peptide PEP27 (VP1 residues 142–156) was recognized as an EV-A71 IgM-specific immunodominant epitope; moreover, synthetic peptide PEP23 (VP1 residues 41–55) was identified as an EV-A71 IgG cross-reactive immunodominant epitope. Jiang et al. [93] utilized the norovirus P protein as a carrier for delivery of the EV-A71 epitope, which is the 71–6 epitope spanning amino acids 176–190 of VP3. Sera from mice immunized with chimeric P protein displaying the 71–6 epitope protected suckling mice against a lethal dose of EV-A71 challenge.

### VLP-based vaccines

VLPs have been applied in the production of other viral vaccines such as hepatitis B virus and human papillomavirus, and could also be a suitable choice for EV-A71 vaccine development. The morphological characteristic and antigenicity of VLPs are similar to those of the naïve virus. Moreover, VLPs are associated with greater safety because they lack the viral genome and thus cannot replicate in the host. However, VLPs can still effectively elicit innate and adaptive immunity.

The baculovirus expression system has been widely used for VLP production. A recombinant baculovirus co-expressing the P1 region and the viral protease 3CD of EV-A71 with different promoters was infected to insect cells for VLPs production (subgenotype C2, *neu* strain) [94, 95]. The viral protease 3CD can cleave the P1 region of structural proteins [VP0 (VP4 and VP2), VP3, and VP1], which is important to constitute the virus capsid. However, this VLP production method suffers from low yields and excessive VLP degradation. Several factors influencing the expression yields of VLP, including the control of various promoters, insect cell types, and incubation time. After researchers serially modified the system for the construction of recombinant baculoviruses, the yield of EV-A71 VLPs improved [96]. EV-A71 VLPs elicited humoral and cellular immune responses in immunized mice, and vaccination of female mice with VLPs, protected the neonatal mice from a lethal dose of EV-A71 challenge [97]. In another study, Macaque monkeys were vaccinated with EV-A71 VLPs produced from baculovirus, which elicited immune responses [98]. In addition to the baculovirus expression system, EV-A71 VLP also can be generated in yeast such as *Saccharomyces cerevisiae* or *Pichia pastoris*, which showed protective efficacy against EV-A71 challenge in mice. In addition, maternal immunization with VLPs could also protect neonate mice against lethal EV-A71 challenge [96, 99].

Chimeric VLPs, including adenovirus or varicella-zoster virus-based VLPs, have also been applied to co-express the P1 and 3CD regions of EV-A71, which could both induce an EV-A71-specific immune response and neutralization antibodies in vaccinated mice, and exhibited protective efficacy against EV-A71 infection [100, 101].

### Live-attenuated vaccines

According to the experience in developing the poliovirus Sabin vaccine, and the numerous advantages of live-attenuated vaccines, including elicitation of long-lasting immunity and cost-effective production, researchers have continued to investigate potential candidates for an EV-A71 live-attenuated vaccine. EV-A71(S1–3′) was derived from the prototype EV-A71(BrCr) strain. Five cynomolgus monkeys were inoculated with EV-A71 (S1–3′) via an intravenous route, followed by challenge with a lethal dose of EV-A71(BrCr-TR), demonstrating induction of an efficient immune response, and the sera showed neutralization activity against EV-A71(BrCr-TR) (subgenotype A) and other subgenotypes, including B1, B4, C2, and C4. However, EV71(S1–3′) caused tremor in the inoculated monkeys, and the virus was isolated from the lumbar spinal cord of inoculated monkeys on days 4 or 10 post-inoculation [102]. Therefore, the safety issue of live-attenuated vaccine remains a concern.

Because the detailed molecular pathogenic mechanism of EV-A71 infection remains unexplored, the virulence determinants of EV-A71 are still being investigated. The amino acid residue 145 in VP1 is considered to be an important factor for EV-A71 virulence and receptor attachment [103, 104]. Mutation of a single amino acid, glutamine (Q) to glutamic acid (E), at residue 145 of VP1 in the subgenotype C4 of EV-A71 was used to generate a mouse-virulent EV-A71 strain [105]. Viruses harboring the VP1–145E mutation could also induce neurological symptoms in cynomolgus monkeys; therefore, VP1–145E viruses are more virulent than VP1–145G viruses in cynomolgus monkeys [103]. The nucleotide 158 in the stem loop II region of the EV-A71 5′ UTR play a pivotal role in EV-A71 virulence. The nucleotide substitution of C158U reduced the translation activity of EV-A71, and attenuated EV-A71 virulence in a mouse model [106].

Moreover, the nucleotide substitutions of G64R, G64 T, and S264 L in EV-A71 3D polymerase were shown to contribute to EV-A71 replication fidelity. Enhancement of the fidelity of 3D polymerase can improve the stability and safety of live-attenuated vaccines [107]. Another study also indicated that EV-A71 with the RdRp-G64R and RdRp-L123F mutations attenuated the virulence of the virus in an AG129 mouse model [108]. Yee et al. [109] constructed an miRNA-based EV-A71 vaccine strain, pIY, which carried let-7a and miR-124a target genes. They found that the viral yield of the pIY strain was much lower than that of the EV-A71 wild-type B4 strain 41 in SHY-5Y cells. Moreover, the pIY strain could still protect mice against EV-A71 in a mouse-adapted strain challenge.

In recent years, a new combination strategy of codon deoptimization and synthetic virus production has emerged for vaccine development. Tsai et al. [110] found that rgEV-A71-CD-HF, a virus with a deoptimized VP1 codon, and a high-fidelity virus with nucleotide substitutions of G64R and L123F in 3D polymerase showed less virulence in a mouse model.

### Mucosal vaccines

The mucosal immune response, which is effectively induced by the administration of a vaccine onto the mucosal surface, is the first line of defense against pathogen invasion. Several mucosal vaccines have been licensed for use in humans, such as oral vaccines against poliovirus, rotavirus, *Vibrio cholera,* and *Salmonella* Typhi, and an intranasal vaccine against influenza virus. The advantages of mucosal vaccines are that they are good inducers of mucosal and systematic immunity, and the needle-free administration is more acceptable for infants and young children [111, 112]. Although the poliovirus Sabin vaccine is a successful example of a mucosal vaccine, the safety issue of live-attenuated vaccine remains a concern. To date, there have been few studies focused on development of a mucosal vaccine for EV-A71. As summarized above, several studies involved immunizing mice with recombinant VP1 protein by an oral route [80–84]; however, these vaccines are still at the preclinical stage of research and validation. Recently, Lin et al. [113] found that the titers of EV-A71-specific IgG and IgA, T-cell proliferative response, and interleukin-17 secretion were increased in a group of BALB/c mice immunized with a CpG-adjuvanted inactivated EV-A71 vaccine via an intranasal route. In addition, this vaccine could also protect human scavenger receptor class B, member 2 transgenic (hSCARB2-Tg) mice against lethal EV-A71 challenge. Although a mucosal vaccine seems to be another good choice for EV-A71 vaccine development, there are still some challenges in mucosal vaccine design to overcome, including how to effectively breach the epithelial barrier, and the relatively large amounts of vaccine needed for mucosal immunization.

## Conclusions and prospects

EV-A71 is one of the most pathogenic enteroviruses infecting humans, with many outbreaks occurring worldwide causes a wide range of human diseases. However, there is still no clinically approved antiviral drug available for the prevention and treatment of the EV-A71 infection. Although the development of antiviral therapy and vaccine represents a major challenge, the progress made so far in understanding the viral replication mechanism has provided novel targets for antiviral therapy and the characterization of compounds with antiviral activity. The development of pan-enteroviruses vaccine and anti-viral drugs is an important and achievable goal in the future.

## Data Availability

Not applicable.

## Abbreviations

5′ UTR: 5′ untranslated region
Anx2: Annexin A2
APOBEC3G or A3G: Apolipoprotein B messenger RNA-editing enzyme catalytic polypeptide-like 3G
CAMS: Chinese Academy of Medical Sciences
CV-A16: Coxsackievirus A16
DC-SIGN: Dendritic cell-specific intercellular adhesion molecule-3 grabbing nonintegrin
DIDS: 4,4′-diisothiocyano-2,2′-stilbenedisulfonic acid
*E. coli*: *Escherichia coli*
EV-A71: Enterovirus A71
HFMD: Hand-foot-and-mouth disease
HS: Heparan sulfate
IDR: Idarubicin
IRES: Internal ribosome entry site
NF-κB: Nuclear factor-kappa B
NHRI: National Health Research Institutes
PSGL-1: P-selectin glycoprotein ligand 1
SCARB2: Scavenger receptor class B member 2
Sinovac: Sinovac Biotech Co., Ltd
Vigoo: Beijing Vigoo Biological Co., Ltd
VLP: Virus-like particle
